# PU.1 is involved in the immune response to *Aspergillus fumigatus* through upregulating Dectin-1 expression

**DOI:** 10.1186/s12879-016-1632-x

**Published:** 2016-06-16

**Authors:** Min Wang, Zhicheng Liu, Chenyang Liu, Ting Wu, Feng Cai, Quan Wang, Xin Su, Yi Shi

**Affiliations:** Department of Respiratory and Critical Care Medicine, Jinling Hospital, Medical School of Nanjing University, 305 East Zhongshan Road, Nanjing, China; Southern Medical University, Guangzhou, China; Department of Respiratory Medicine, BenQ Medical Center, Nanjing, China

**Keywords:** PU.1, Dectin-1, *Aspergillus fumigatus*, Immunity

## Abstract

**Background:**

Invasive aspergillosis is a life-threatening disease, and its incidence has increased in the recent past. Dectin-1 recognizes β-glucans and mediates innate immune responses to *Aspergillus fumigatus*. Transcription factor PU.1 has been the focus of recent research due to its role in inflammation and infection. However, its role in Dectin-1 regulation during *A. fumigatus* infection remains to be elucidated.

**Methods:**

THP-1 cells were stimulated with *A. fumigatus* conidia. We then used real-time RT-PCR, Western blot, and immunofluorescence assays to analyze the mRNA and protein levels and cellular distribution, respectively, of Dectin-1 and PU.1 in stimulated THP-1 cells. Additionally, we used the luciferase reporter assays, chromatin immunoprecipitation (ChIP) assays, electrophoretic mobility shift assays (EMSA), and RNA interference experiments to investigate the role of PU.1 in Dectin-1 regulation.

**Results:**

Our results revealed that Dectin-1 mRNA and protein levels as well as the PU.1 protein level were increased in THP-1 cells stimulated with *A. fumigatus* conidia, while the mRNA expression level did not significantly change between the stimulated and control groups. We also observed that PU.1 translocated into the nucleus in stimulated THP-1 cells. The results of the luciferase reporter assay showed that PU.1 promoted human Dectin-1 (hDectin-1) gene activity. ChIP and EMSA indicated that PU.1 could bind with hDectin-1 gene promoter at three potential transcription factor-binding sites (TFBSs). In addition, knockdown of PU.1 significantly decreased Dectin-1 expression.

**Conclusions:**

This study demonstrated the novel role of PU.1 in the immune response to *A. fumigatus* through upregulation of Dectin-1 expression and its translocation to the nucleus in *A. fumigatus*-stimulated THP-1 cells.

## Background

Invasive aspergillosis (IA) is a life-threatening disease whose incidence is growing worldwide due to the increasing number of immunocompromised patients over the last decades [1]. Despite therapeutic advances and better diagnostic tools and criteria, IA remains difficult to diagnose and treat, and the mortality associated with this condition remains high, ranging from 60 % to 90 % [2]. *Aspergillus fumigatus* is a ubiquitous opportunistic fungal pathogen that accounts for more than 90 % of all cases of IA [3].

The innate immune system serves as the first line of defense against fungal infection, as it is capable of recognizing and initiating an effective response to eliminate invading *A. fumigatus* [4]. However, there is limited knowledge of the regulation and detailed immune mechanism involved in the defense against *A. fumigatus* infection. Therefore, further investigation of the mechanism would help develop effective immunoprophylaxis and to improve patient prognosis.

Pattern recognition receptors (PRRs) recognize pathogen-associated molecular patterns (PAMPs) expressed in the cell wall during fungal infection [4, 5]. C-type lectin receptors (CLRs) are a large family of receptors that bind to carbohydrates in a calcium-dependent manner. They are expressed by several cell types such as macrophages and dendritic cells (DCs), which phagocytose various glycoproteins and microbes for clearance and antigen presentation to T lymphocytes. CLRs are widely recognized to mediate innate immune responses to fungi, with Dectin-1 being the best characterized receptor in the context of fungal infections [5]. Dectin-1 initiates antifungal immunity by recognizing the β-glucan present in the cell walls of *A. fumigatus* [6, 7].

PU.1, a member of the E-twenty-six (ETS) family, is a transcription factor that is essential for the development of hematopoietic stem cells and regulation of the immune response [8, 9]. In addition, PU.1 participates in regulating the expression of a variety of PRRs, including toll-like receptor (TLR)-4 [10], TLR-9 [11], mannose receptor (MR) [12], a dendritic cell-specific C-type lectin (DC-SIGN) [13], and Dectin-1 [14, 15]. In a previous study, pneumocystis-infected mice exhibited reduced Dectin-1 and PU.1 expression levels decreases in alveolar macrophages (AMs) [15]. However, another study incubated human bronchial epithelial cells (HBE) with *A. fumigatu*s and found that Dectin-1 was upregulated in these cells [16]. Despite these studies, the role of PU.1 in regulating the transcription and expression of the hDectin-1 gene during *A. fumigatus* infection remains unclear. Therefore, we investigated the role of PU.1 regulating Dectin-1 expression in THP-1 cells stimulated with *A. fumigatus*. Additionally, we characterized the transcription factor-binding sites (TFBSs) in the hDectin-1 gene promoter, indicated the regulation of hDectin-1 gene via PU.1, and further determined changes in the expression of Dectin-1 following knockdown of PU.1.

## Methods

### Cells, reagents, and antibodies

THP-1 (human monocytic leukemia) cells were cultured in RPMI-1640 medium (Gibco; Invitrogen, Carlsbad) and human embryonic kidney (HEK)-293 T cells were cultured in Dulbecco’s modified Eagle’s medium (DMEM; Gibco). These two cell lines were gifts from Prof. Xing (Laboratory of Immunology and Virology, Nanjing University, China) [17]. Both the culture mediums were supplemented with 10 % fetal bovine serum (FBS; Hyclone, Thermo Scientific, Logan), and 1 % antibiotic-antimycotic solution (Gibco). The cells were cultured at 37 °C in an atmosphere containing 5 % CO_2_. Polymerase chain reaction (PCR) primers were synthesized by GenScript (Nanjing, China), while probes for EMSA were synthesized by Viagene Biotech (Ningbo, China). All the primers and probes used in this study are listed in Table 1, 2 and 3. The following antibodies were used for Western blot analysis: rabbit anti-PU.1, rabbit anti-β-tubulin (Cell Signaling Technology, Danvers); goat anti-Dectin-1 (N-16, Santa Cruz Biotechnology, Santa Cruz); mouse anti-GAPDH, mouse anti-β-actin, and rabbit anti-Histone (Abmart, Shanghai, China). Other secondary antibodies were purchased from Cell Signaling Technology. Rabbit anti-PU.1 (H-135, Santa Cruz Biotechnology) and rabbit IgG (Upstate Biotechnology, Waltham) were used in ChIP. Antibodies for Alexa Fluor-488 donkey anti-rabbit IgG and Alexa Fluor-568 donkey anti-goat IgG were purchased from Life Technologies.

**Table 1:** Primer sequences for real-time PCR

Primers	Sequence (5′–3′)
hDectin-1-Forward	GCAATACCAGGATAGCTGTTG
hDectin-1-Reverse	CCAAGCATAGGATTCCCAA
hPU.1- Forward	CGTGCACAGCGAGTTCGA
hPU.1-Reverse	GCTCTGGAGCTCCGTGAAGT
GAPDH- Forward	ACAGTCAGCCGCATCTTCTT
GAPDH-Reverse	ACGACCAAATCCGTTGACTC

**Table 2:** Primer sequences for PCR of ChIP

Primers	Sequence (5′–3′)
hDectin-1-A- Forward	CTTTTCCTTAGCAAACTCCACT
hDectin-1-A-Reverse	TCAGTAATACTATTGCAACAGGA
hDectin-1-B- Forward	CCTTCCATAAGCTGTTCCTTGCA
hDectin-1-B-Reverse	CAAGGAATCAAATCAAAGCTGACTC
hDectin-1-C- Forward	GCCTACTATATAGACTTGGAAAAG
hDectin-1-C-Reverse	CTTTGGGAATTATCAGATATTAACAC
hDectin-1-D- Forward	AGCCGAGATCGTGCCATTTGCA
hDectin-1-D-Reverse	TGCTTCTCAAAGGGCAGAGAAAGA
hDectin-1-E- Forward	GAGTCAGCTTTGATTTGATTCCTTG
hDectin-1-E-Reverse	ACTTTTCCAAGTCTATATAGTAGGC

**Table 3:** Probe sequences for EMSA

Probes	Sequence (5′–3′)	Biotin	Gene**s**
Positive control	PU.1-TP CTGCCTCCTACTTCTCCTTTTCTGC PU.1-BM GCAGAAAAGGAGAAGTAGGTGGCAG	+	CD11b
Probe A	probe1-TP TTACTTTTGGGGAAGTTGAGTTCAG probe1-BM CTGAACTCAACTTCCCCAAAAGTAA	+	Dectin-1
Probe B	probe1-BM CTGAACTCAACTTCCCCAAAAGTAA probe2-TP TGATGAAAGAGGCAGTGTAGCGTAA	+	Dectin-1
Probe C	probe3-TP GATTGAACAAGAAAATATGTACAAC probe3-BM GTTGTACATATTTTCTTGTTCAATC	+	Dectin-1
Competitor	Paired with each biotin-labeled probe	-	

### *A. fumigatus* strain and stimulation with conidia

*A. fumigatus* A1 was kindly provided by the Microbiological Laboratory of Jinling Hospital. After culture on Sabouraud dextrose agar plates for 7 days at 28 °C, conidia were harvested by washing the plates with sterile phosphate-buffered saline (PBS)-0.1 % (vol/vol) Tween 20 (PBST). The suspension was then gently filtered through a 40-μm cell strainer to separate conidia from mycelium. Then, the conidia were thoroughly washed, centrifuged, and resuspended in sterile PBST at concentrations ranging from 5.0 × 10^7^ to 1.0 × 10^8^ colony-forming units (CFU)/mL, and stored at 4 °C within 2 weeks for further experiments. The THP-1 cells were cultured in RPMI-1640 medium at a density of 1.0–1.5 × 10^6^/mL, and stimulated with *A. fumigatus* conidia at a multiplicity of infection (MOI) of 1.

### Quantitative real-time RT-PCR

The total RNA precipitated from the lysates of THP-1 cells stimulated with *A. fumigatus* conidia and the lysates of unstimulated THP-1 cells were used for reverse transcription (RT) with a Primescript RT reagent kit (R036A; Takara, Tokyo). The mRNA expression levels were analyzed by real-time PCR, as described previously [18]. The specificity of primers was confirmed by melting curve analysis. The relative expression values were standardized by an internal glyceraldehyde-3-phosphate dehydrogenase (*GAPDH*) control. The fold changes of mRNA were calculated according to the formula 2 ^(Δ*CT* of gene – Δ*CT* of GAPDH)^, where *C*_*T*_ is the threshold cycle.

### Western blot analysis

THP-1 cell lysates of the mock or *A. fumigatus* conidia-stimulated cells were subjected to sodium dodecyl sulfate-polyacrylamide gel electrophoresis (SDS-PAGE) and transferred to polyvinylidene difluoride (PVDF) membranes (Millipore, Billerica), and then incubated with primary antibodies overnight. After washing the membranes, they were incubated with horseradish peroxidase (HRP)-conjugated secondary antibodies for 90 min at room temperature. Images were captured using Tanon Imaging System (Tanon, Shanghai, China). For the fractionation of lysates, stimulated or control cells were washed, and cytoplasmic and nuclear fractions were isolated using cytoplasmic/nuclear protein extraction kit (KeyGene Biotechnology, Nanjing, China). The fractions were processed as described above for immunoblot analysis.

### Immunofluorescence microscopy

Stimulated and unstimulated THP-1 cells were washed twice with PBS at various time points, fixed with 4 % paraformaldehyde for 30 min, and permeabilized with 0.1 % Triton X-100 for 10 min, and then washed with PBS. After blocking with 5 % bovine serum albumin (BSA) in PBS for 60 min at 37 °C, the cells were incubated with both rabbit anti-PU.1 and goat anti-Dectin-1 antibodies (1:100 dilutions for both antibodies) at 4 °C overnight. The cells were washed with PBST, and then incubated with Alexa Fluor-488 donkey anti-rabbit IgG and Alexa Fluor-568 donkey anti-goat IgG (1:200 dilution for both antibodies) at 37 °C for 1 h. Then, the cells were washed and stained with 1 μg/mL of 4′,6-diamidino-2-phenylindole (DAPI). After washing, the slides were carefully observed under a confocal microscope (Carl Zeiss Jena, Oberkochen).

### Plasmid constructs and luciferase reporter assays

The 2.0-kb DNA fragment containing the Dectin-1 gene promoter was PCR amplified from human genomic DNA using the following primers: 5′-GCGGTACCGCAGATCGAGACCATTCTGGTTAACAC-3′ and 5′-GCAAGCTTGCGAAACTATGCTGTGGTAATTTT-3′. Plasmid pGL3-Decin-1 was constructed by cloning the amplified fragment into the firefly luciferase reporter vector pGL3 (Promega, Madison). The PU.1 expression plasmid pRK5-PU.1 was obtained by cloning human PU.1 cDNA into EcoRI-digested pRK5-HA (Promega) and a selection of plasmids with properly oriented inserts. HEK-293 T cells were seeded in 24-well plates in total DMEM until 50–70 % confluence was achieved. The cells were then transfected with pRK5-HA or with various amounts of pRK5-PU.1, along with pGL3-Dectin-1 or pGL3-basic, and pRL8-SV40 by using Lipofectamine 2000 (Invitrogen). The total amount of DNA was maintained at a constant level by adding empty control plasmid. At 24 h post-transfection, the cells were stimulated by 10 ng/mL of phorbol-12-myristate-13-acetate (PMA) for 12 h, and cell lysates were analyzed for the activities of firefly and Renilla luciferases (Promega) according to the manufacturer’s instructions.

### Chromatin immunoprecipitation assays

ChIP assays were performed using EZ-ChIP™ kit (Upstate Biotechnology) according to the manufacturer’s instructions. In short, 1–1.5 × 10^7^ THP-1 cells were stimulated by 10 ng/mL PMA for 2 h, cross-linked with 1 % formaldehyde, and lysed in SDS lysis buffer. The chromatin samples were sonicated at high power for 7 min (in cycles of 5-s pulses with 10-s pause) using a sonicator (Bioruptor™; Diagenode, Belgium). After preclearing with salmon sperm DNA/Protein A-agarose, the protein extracts were subjected immunoprecipitated with antibodies against PU.1 (H-135), RNA polymerase, and normal rabbit IgG. The resultant immune complexes were extensively washed, and the DNA was recovered by phenol:chloroform extraction and resuspended in 50-μL volume. PCR amplification was performed by using the primers listed in Table 2. The resultant PCR products were analyzed by 2 % agarose gel electrophoresis.

### Electrophoretic mobility shift assays (EMSA)

THP-1 cells were plated on RPMI-1640 at a density of 1.0–1.5 × 10^7^ in the presence of 10 ng/mL PMA, and collected after 2 h. Nuclear extracts from PMA-stimulated and control cells were prepared using nuclear protein extraction kit (KeyGene Biotechnology). The probes used for EMSA are listed in Table 3. Briefly, 3 μg of nuclear protein samples were incubated with 10× binding buffer, 1.0 μg/μL poly (dI-dC), and 0.5 μL biotin-labeled probes in an EMSA kit (Viagene Biotech). Where indicated, 0.83 μL or 3.33 μL of specific, cold-competitor probe in 10× or 100× competing buffer was added before the biotin-labeled probe. The incubation mixtures were separated by 6.5 % non-denaturing PAGE, and the bands were detected by autoradiography.

### RNA interference experiments

PU.1-specific small interfering RNA (siRNA) or a nonspecific siRNA control (siCtrl) were chemically synthesized at GenePharma (Shanghai, China). The sequence of the PU.1 siRNA was as follows: 5′-UAUAGAUCCGUGUCAUAGGGCACCA-3′. A 5-μL of aliquot of 20 μM siRNA was introduced into 1 × 10^6^ THP-1 cells by using Lipofectamine 2000, according to the manufacturer’s instructions. The transfected cells were incubated for 24 h*,* and the cells were harvested and subjected to real-time PCR and Western blot analyses to detect PU.1, Dectin-1, and the control cell populations.

### Statistical analysis

A two-tailed Student’s paired *t* test was used to evaluate the data by SPSS software (IBM SPSS, Armonk, USA), and values of *p* < 0.05 were considered to be statistically significant.

## Results

### Dectin-1 and PU.1 mRNA and protein expression levels in *A. fumigatus* conidia*-*stimulated THP-1 cells

**Fig. 1:**
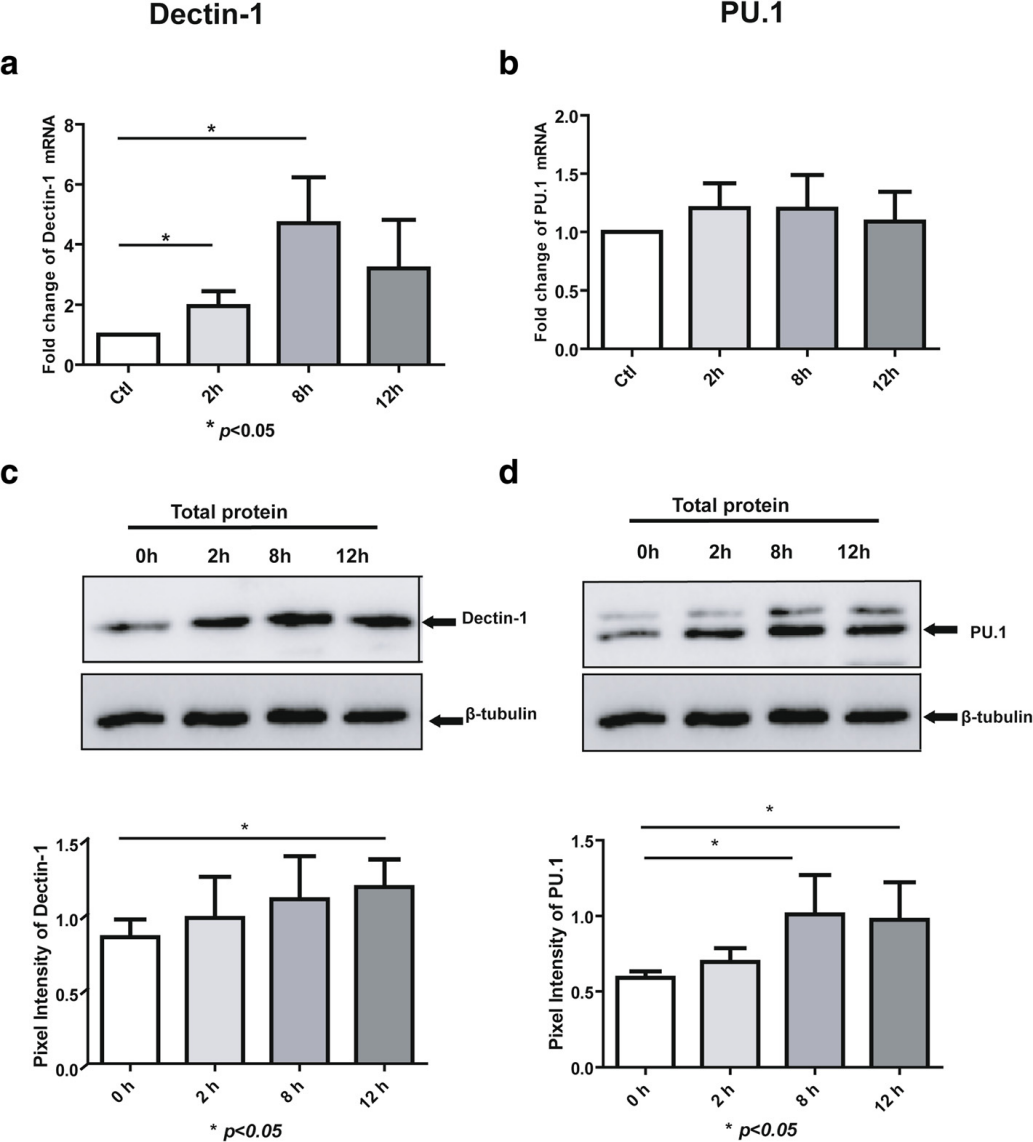
Expression of Dectin-1 and PU.1 in *A. fumigatus* conidia-stimulated THP-1 cells. Total RNA were prepared from the stimulated or unstimulated cells at various time points, and real-time PCR was performed to measure the mRNA levels of Dectin-1 (**a**) and PU.1 (**b**) at 2, 8, and 12 h after stimulation, respectively. Induction of Dectin-1 and PU.1 expression. Cell lysates were prepared and subjected to SDS-PAGE and Western blot analysis with antibodies against Dectin-1 (**c**), PU.1 (**d**), or β-tubulin at the indicated time points. The ratio of Dectin-1 and PU.1 pixel intensity to β-tubulin were analyzed by Image J software (Version 1.39u). The experiments were repeated at least thrice, and the data from one representative experiment with two technical repeats are presented (*, *p* < 0.05)

In order to study the PU.1 and Dectin-1 expression patterns in the antifungal immune response, THP-1 cells were exposed to *A. fumigatus* conidia at an MOI of 1 for 2, 8, and 12 h. Real-time PCR and Western blot analyses were then used to analyze the Dectin-1 and PU.1 mRNA and protein expression levels, respectively, in THP-1. It was observed that both Dectin-1 mRNA and protein levels were upregulated in the conidia*-*stimulated THP-1 cells compared to the controls (Fig. 1a and c). Interestingly, the results also found that PU.1 mRNA levels remained unchanged in THP-1 cells; however, the PU.1 protein expression was higher in the stimulated cells than in the control cells (Fig. 1b and d).

### Cellular localization of Dectin-1 and PU.1

**Fig. 2:**
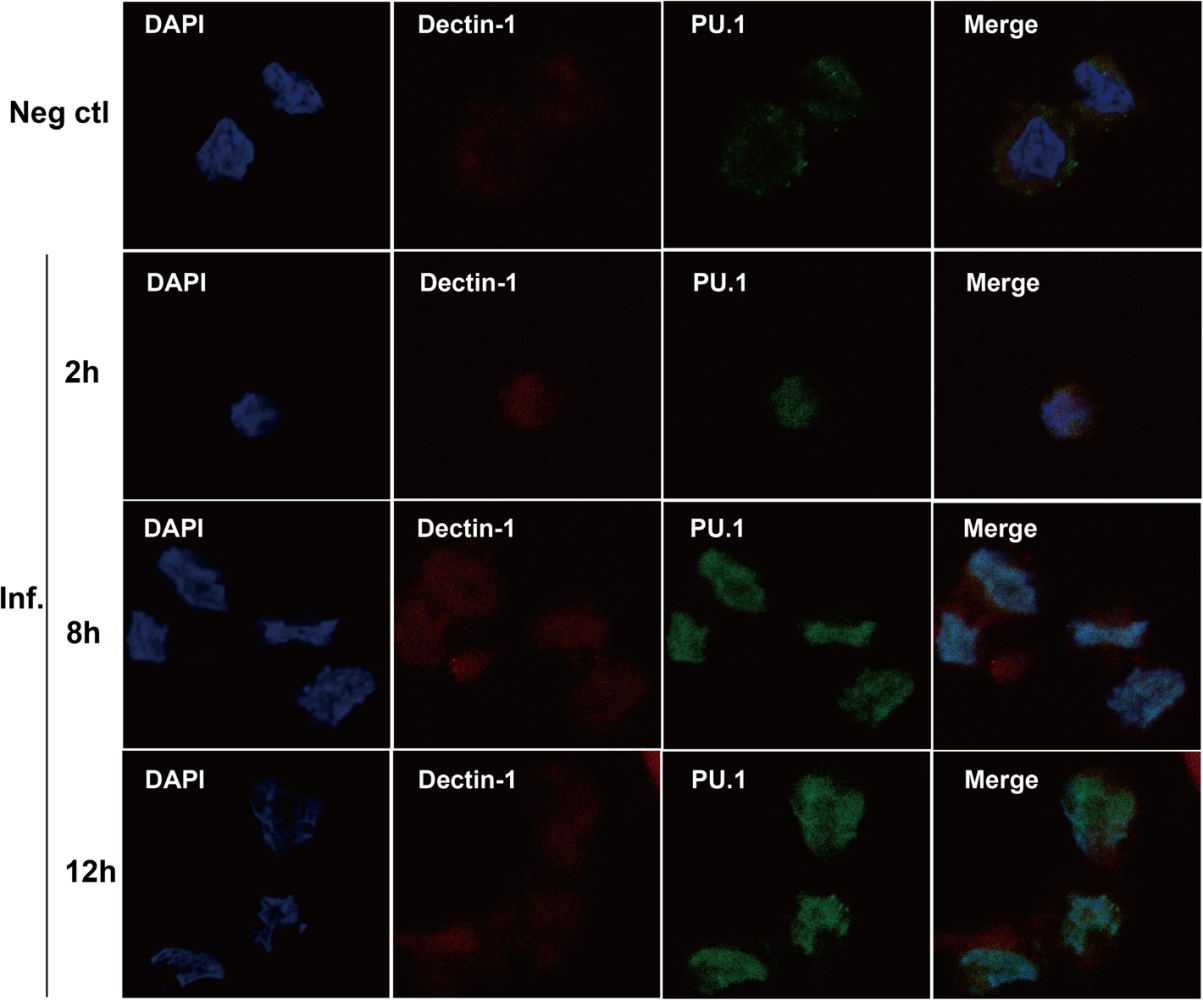
Immunofluorescence staining for Dectin-1 and PU.1 expression and location in *A. fumigatus* conidia-stimulated THP-1 cells. THP-1 cells were untreated (control) or incubated with *A. fumigatus* conidia for 2, 8, or 12 h. After permeabilization with Triton X-100, the cells were incubated with goat anti-Dectin-1 and rabbit anti-PU.1 antibodies at a 1:100 dilution, stained with Alexa Fluor-568 donkey anti-goat IgG and Alexa Fluor-488 donkey anti-rabbit IgG at 1:200 dilution, counterstained with DAPI, and analyzed by confocal microscopy

We performed immunofluorescence analysis to the cellular localization of Dectin-1 and PU.1 in THP-1 cells. The stimulated (MOI = 1) or unstimulated THP-1 cells, fixed at different time points after stimulation (2, 8, and 12 h), were stained with diluted goat anti-Dectin-1 and rabbit anti-PU.1 antibodies. In the confocal study, Dectin-1 protein (red) was dispersed in the membrane of conidia-exposed THP-1 cells (Fig. 2). Additionally, Dectin-1 protein expression increased with time of exposure (Fig. 2), as explained previously (Fig. 1c). Meanwhile, translocation of PU.1 protein (green) into the nucleus was markedly stimulated by *A. fumigatus* conidia (Fig. 2), which was not the case in the control cells.

### *A. fumigatus* activates PU.1 nuclear translocation

**Fig. 3:**
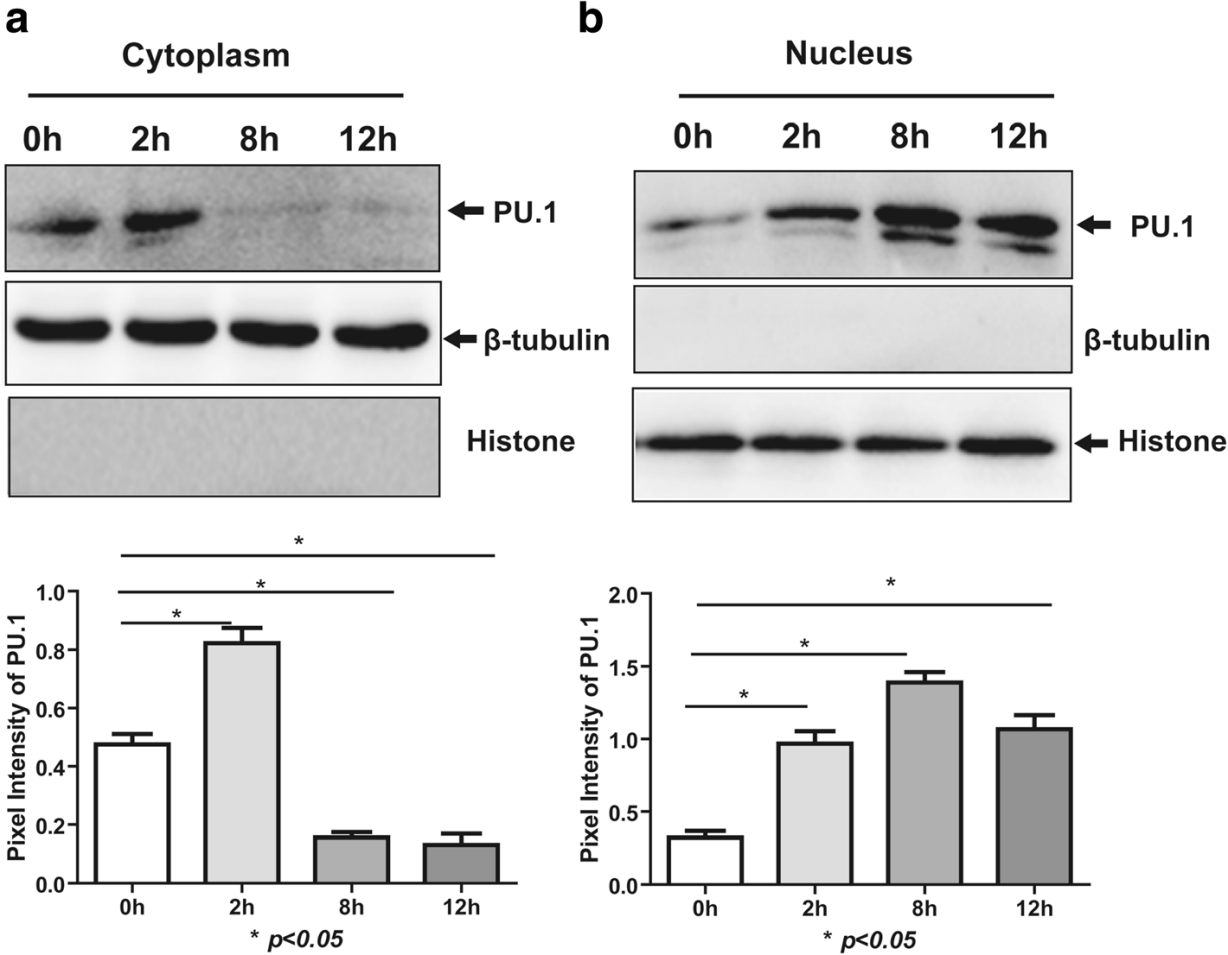
Effect of *A. fumigatus* on PU.1 translocation. THP-1 cells were stimulated with *A. fumigatus* at an MOI of 1. At 2, 8, and 12 h after stimulation, cell lysates were separated into cytoplasmic and nuclear fractions. Both cytoplasmic (**a**) and nuclear proteins (**b**) were analyzed by SDS-PAGE and immunoblotting with anti-PU.1 antibodies to reveal the localization of PU.1. Nucleus-specific anti-Histone antibodies and cytoplasmic-specific anti-β-tubulin antibodies were used as controls. The experiments were repeated at least thrice, and the data from one representative experiment with two technical repeats are presented (*, *p* < 0.05)

To gain further insights into the role of *A. fumigatus* in the activation of PU.1, we studied nuclear translocation by Western blot analysis of the cytoplasmic and nuclear cell fractions. In the cytoplasm, the PU.1 protein level started to decrease at 8 h after stimulation (Fig. 3a). Meanwhile, in the nucleus, the PU.1 protein levels were higher compared to the corresponding levels in the control cells; moreover, the PU.1 protein levels in the nuclear lysates of the stimulated cells increased with time (Fig. 3b). The histone and β-tubulin protein levels in the cytoplasmic and nuclear fractions were similar in the stimulated and control cells.

### PU.1 promotes hDectin-1 gene activity

**Fig. 4:**
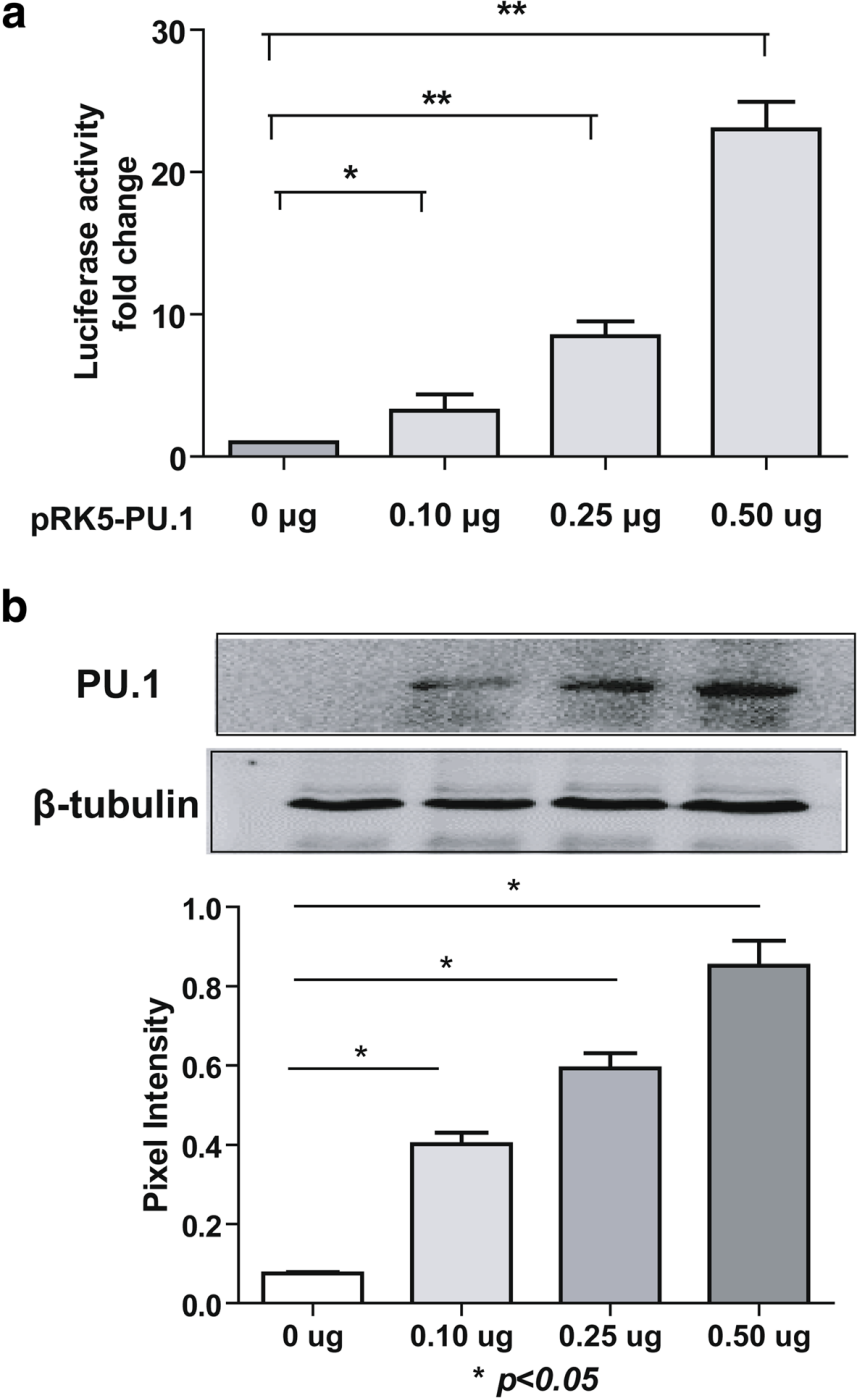
Effect of PU.1 on Dectin-1 activation. **a** HEK-293 T cells were cotransfected with the indicated amount of pRK5-PU.1, 1 μg pGL3-hDectin1, and 10 ng pRL8-SV40 plasmids, and pGL3-basic and pRK5-HA were used as the negative controls. At 24 h post-transfection, the cells were harvested, lysed, and assayed for luciferase activity. The resultant ratios were normalized to the fold change values obtained from HEK-293 T cells cotransfected with pGL3-basic and pRK5-HA. **b** Cell lysates were analyzed by immunoblotting with rabbit anti-PU.1 antibodies to detect the expression of pRK5-PU.1. β-Tubulin was used to verify equal loading of proteins in each lane. The experiments were repeated at least thrice, and the data from one representative experiment with two technical repeats were presented (*, *p* < 0.05)

To investigate whether PU.1 accounts for the hDectin-1 gene promoter activity, HEK-293 T cells were cotransfected with the reporter plasmid pGL3-hDectin-1 and the PU.1-expressing plasmid pRK5-PU.1. HEK-293 T cells were chosen for this experiment instead of monocytes because they normally do not express PU.1; therefore, the influence of endogenous PU.1 can be avoided. Results of the immunoblotting analyses demonstrated the presence of PU.1 protein in transfected cell lysates, indicating that pRK5-PU.1 plasmid could successfully express PU.1 (Fig. 4b). As shown in Fig. 4a, luciferase activity of HEK-293 T cells transfected with 0.10, 0.25, and 0.50 μg of pRK5-PU.1 increased by 3.2–, 8.5–, and 23.0-fold that of the control, respectively, indicating that PU.1 activated hDectin-1 expression in a dose-dependent manner.

### PU.1 binds directly to the hDectin-1 gene promoter

**Fig. 5:**
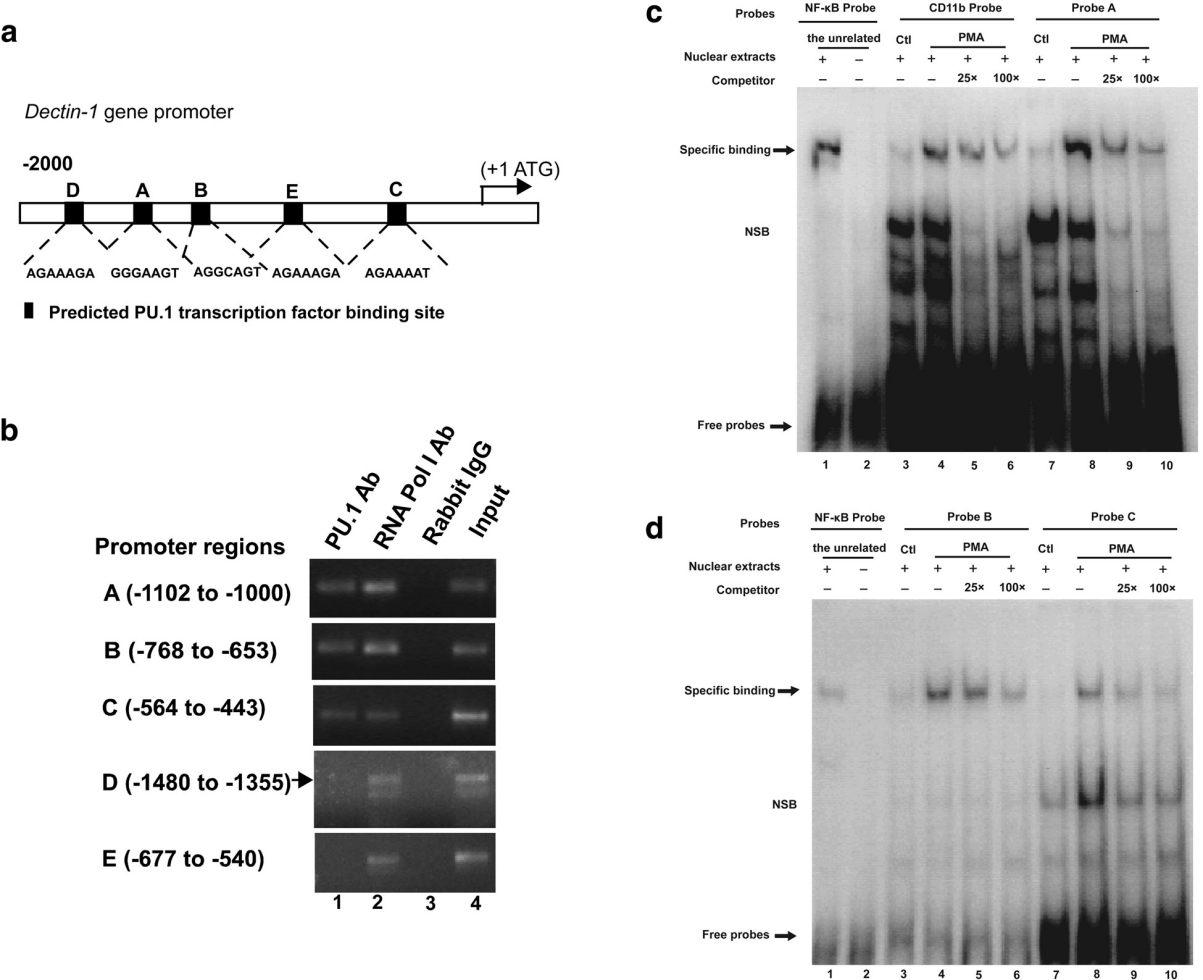
Binding of PU.1 to the hDectin-1 gene promoter. **a** Predicted PU.1 transcription factor-binding sites (TFBSs) within the proximal 2000 bp of the hDectin-1 gene promoter were identified. Potential binding sites were analyzed, including A (GGGAAGT, –1053 to –1047), B (AGGCAGT, –719 to –713), C (AGAAAAT, –527 to –521), D (AGAAAGA, –1411 to –1405), and E (AGAAAGA, –618 to –612). **b** Chromatin immunoprecipitation (ChIP) assays of PU.1-binding sites. Chromatins from THP-1 cells were prepared. Shown are the PCR products from: Lane 1, Chromatin incubated with rabbit anti-PU.1 antibodies (H-135); Lane 2, Chromatin incubated with positive control (anti-RNA Polymerase antibody); Lane 3, Chromatin incubated with negative control (normal rabbit IgG); Lane 4, Chromatin before immunoprecipitation (input chromatin). (**c**, **d**) Electrophoretic mobility shift assays (EMSA). Nuclear extracts from THP-1 cells cultured at control conditions or in the presence of PMA were subjected to EMSA. The use of biotin-labeled oligonucleotide probes A, B, and C, carrying the hDectin-1 gene promoter regions A (–1053 to –1047), B (–719 to –713), and C (–527 to –521), respectively, allowed to assess the specificity of the protein-DNA binding. The CD11b probe, which corresponded to the –22 to –12 bp PU.1 consensus sequence located on the CD11b promoter, was the positive control. Competitors were unlabeled oligonucleotide probes, and their concentrations were 25-fold and 100-fold. The NF-kB probe was the input probe, and the unrelated were nuclear extracts with active NF-kB protein. Specific binding: protein-DNA complexes of PU.1 or NF-kB proteins and the probes. NSB: nonspecific binding. Free probes: free biotin-labeled oligonucleotide probes that did not bind to the proteins. The experiments were repeated at least thrice, and the data from one representative experiment with two technical repeats are presented (*, *p* < 0.05)

To investigate the regulation of hDectin-1 gene expression, we analyzed the 2000-bp region upstream from the transcription initiation start site of the hDectin-1 gene promoter for candidate TFBSs using TRANSDAC [19] and JASPAR [20]. A total of five potential TFBSs were identified for PU.1 (Fig. 5a), which including A (GGGAAGT, –1053 to –1047), B (AGGCAGT, –719 to –713), C (AGAAAAT, –527 to –521), D (AGAAAGA, –1411 to –1405), and E (AGAAAGA, –618 to –612).

The results of the luciferase reporter assays indicate that PU.1 activates the hDectin-1 gene promoter (Fig. 4a). We performed ChIP to determine whether PU.1 interacts with the hDectin-1 gene promoter in THP-1 cells (Fig. 5b). To verify the five putative PU.1 binding motifs, the DNA fragments were amplified with specific hDectin-1 gene-targeting primers (Table 2). The results showed that three regions relative to the transcription start site (+1) of the hDectin-1 gene: –1102 to –1000, –768 to –653, and –564 to –443 were co-precipitated by PU.1, confirming that PU.1 can bind with hDectin-1 gene promoter.

To further verify the ChIP results, EMSA was performed by using nuclear extracts from THP-1 cells. Three biotin-labeled double-stranded oligonucleotide probes (Table 3) carrying the hDectin-1 gene promoter regions A (–1053 to –1047), B (–719 to –713), and C (–527 to –521) were designed and synthesized. Meanwhile, the PU.1-binding sequence from the CD11b gene promoter [21, 22] was chosen as the positive control. Each of these four probes had an unlabeled oligonucleotide probe as the competitor, and the competitor concentrations were 25-fold and 100-fold. An NF-kB probe was used to verify whether the EMSA system worked properly. PMA activates the protein kinase C (PKC) pathway by mimicking diacylglycerol, a natural ligand and activator of PKCs [23], and can alter gene expression via the activation of PKC and modulating the activity of transcriptional factors that bind cis elements such as NF-kB [24], AP-1 [25], and AP-2 [26]. As only active transcription factors could bind to the specific probe, so we prepared two groups of nuclear extracts: one from THP-1 cells that were treated with PMA for 2 h [27] and one group from untreated cells. As shown in Fig. 5c, Probe A bound to nuclear extracts from PMA-stimulated THP-1 cells (Fig. 5c, lane 8), and the band was located at the same position with the CD11b probe (Fig. 5c, lane 4), indicating the specificity of this protein-DNA complex. Adding increasing amounts of competitors (25- and 100-fold) into the assay resulted in a decreasing intensity of PU.1, demonstrating its presence in this complex (Fig. 5c, lanes 9 and 10). These data suggest that the PU.1 protein directly binds to the –1053 to –1047 gene promoter region. A specific PU.1 band could be observed in EMSA using probes B and C (for the –719 to –713 and –527 to –521 regions, respectively Fig. 5d). These results indicated that PU.1 recognizes and binds to the –1053 to –521 region of the hDectin-1 gene promoter through at least three different sites.

### Effect of PU.1 knockdown on hDectin-1 gene expression

**Fig. 6:**
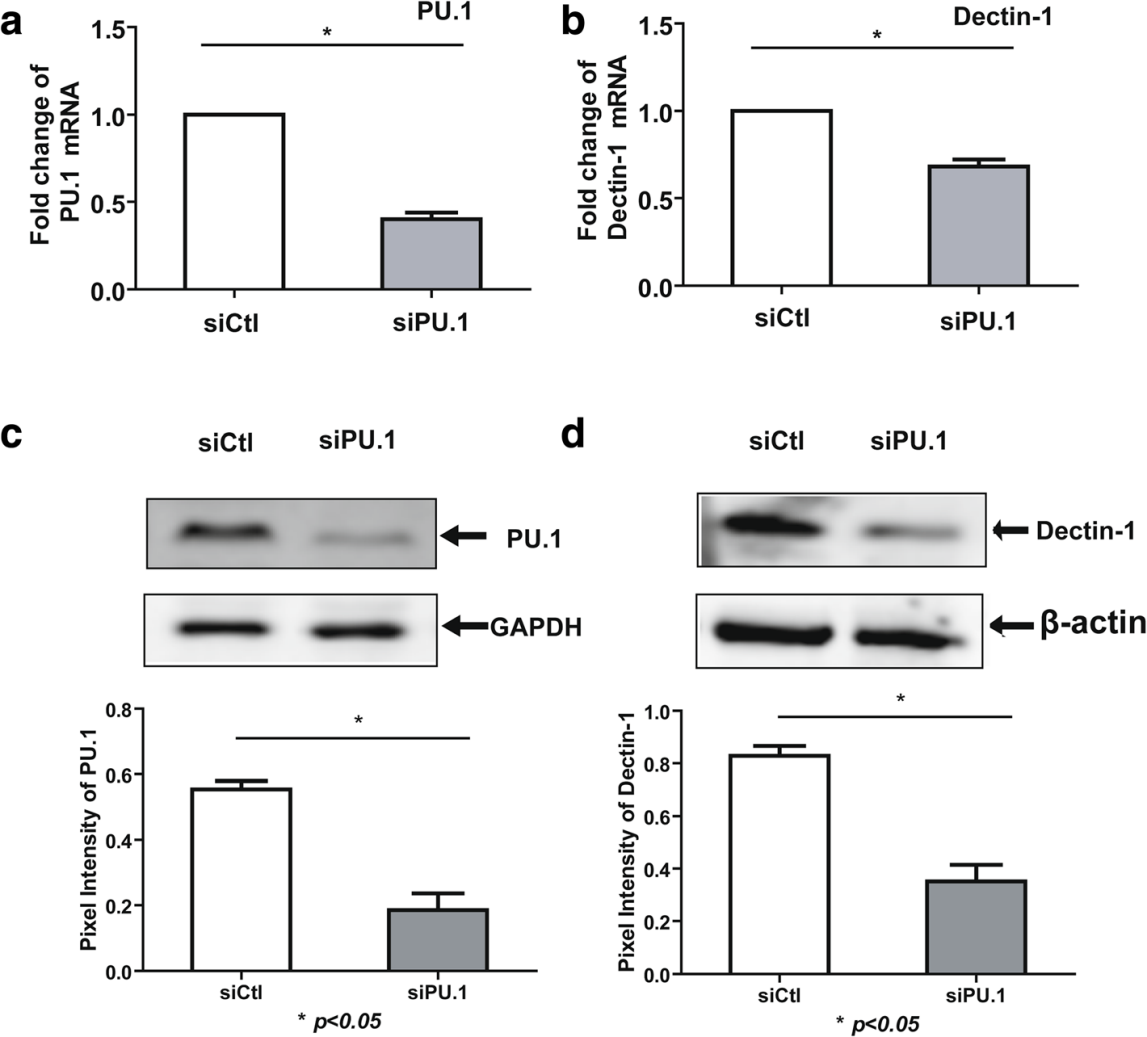
Effect of PU.1 siRNA on Dectin-1 expression in THP-1 cells. THP-1 cells were treated with control siRNA or PU.1 siRNA using Lipofectamine 2000 at the indicated concentrations. Transfected cells were incubated for 24 h*.* The cells were harvested and subjected to real-time PCR and Western blot analysis for the detection of PU.1 (**a**, **c**), Dectin-1 (**b**, **d**), or controls. Data are the means ± SD of three experiments (*, *p* < 0.05)

In order to further confirm that PU.1 regulates the expression of the hDectin-1 gene, THP-1 cells were transfected with control or PU.1 siRNA, and assayed for the expression levels of PU.1 and Dectin-1. Transfection with PU.1 siRNA resulted in approximately 60 % knockdown of PU.1 mRNA expression (Fig. 6a) relative to the control siRNA. In addition, PU.1 silencing decreased Dectin-1 mRNA expression by approximately 32 % in THP-1 cells relative to the expression level in the controls (Fig. 6b). Results of the Western blot analysis also confirmed that THP-1 cells transfected with PU.1 siRNA showed a significant reduction in the Dectin-1 protein expression (Fig. 6c and d), but cells transfected with control siRNA showed no such reduction. These results indicate that PU.1 may participate in the regulation of hDectin-1 gene expression in THP-1 cells.

## Discussion

Dectin-1 is a member of the CLR family and is expressed on the surface of many cells [28]. Its involvement in the immune defense against *A. fumigatus* has been widely confirmed [29]. In our previous study, *A. fumigatus* was shown to induce the upregulation of Dectin-1 in HBE cells in a TLR2-dependent manner [16]. In the present study, we found that Dectin-1 mRNA and protein expression levels were increased in THP-1 cells stimulated with *A. fumigatus* conidia.

PU.1 belongs to the ETS family and has a great impact on the immunity, primarily through its control of immune cell development [30]. As PU.1 has been the focus of increasing attention for its role in inflammation and infection [31–33], we evaluated PU.1 expression at the mRNA and protein levels in THP-1 monocyte cells exposed to *A. fumigatus* conidia. The results of the present study demonstrated for the first time that the PU.1 protein expression was increased in THP-1 cells incubated with *A. fumigatus* conidia compared to the control cells, while the mRNA level showed no significant differences between the exposed and control cells.

In our study, the PU.1 mRNA level was not correlated with its protein level. Similar to our finding, a previous study reported that the PU.1 mRNA level in preadipocytes was high, while the PU.1 protein is not expressed in these cells [34]. Regulation of gene expression is a complicated and meticulous process, and it is controlled at multiple levels, including transcriptional, post-transcriptional, translational, and post- translational levels [35]. MicroRNAs are a large class of small, noncoding RNAs in plants and animals, and have been known to mediate post-transcriptional regulation [36]. PU.1 is a direct target for miR-155 and the target sequence for miR-155 is localized in the 3′-untranslated region (UTR) of PU.1. Also, miR-155 overexpression in THP-1 cells has been found to decrease the PU.1 protein level [13]. Further studies are required to investigate the precise mechanisms responsible for the different patterns of PU.1 mRNA and protein expression during stimulation with *A. fumigatus*.

In our study, PU.1 protein was localized in the cytoplasm of unstimulated THP-1 cells, and translocated into the nucleus following exposure to *A. fumigatus* conidia. To the best of our knowledge, this is the first study to illustrate the translocation of PU.1 stimulated by *A. fumigatus* conidia. Previously, in HeLa and RAW264.7 macrophage cell models, PU.1 has been shown to enter the nucleus via passive diffusion and active transport. The latter can be facilitated by: (i) the classical pathway requiring importin α and β, (ii) the non-classical pathway requiring only importin β, or (iii) direct interaction with nucleoporins [37, 38]. However, to date, little is known regarding the mechanisms of PU.1 nuclear transport during *A. fumigatus* stimulation. Thus, future studies are necessary to investigate the mechanisms by which *A. fumigatus* drives the transport of PU.1 from the cytoplasm to the nucleus.

The regulation of Dectin-1 expression has not been extensively studied to date; however, downregulation of PU.1 is known to result in decreased Dectin-1 expression in AMs in *Pneumocystis*-infected mice [15]. In another study, BLT1 was found to modulate the transcription of Dectin-1 via a GM-CSF/PU.1 cascade [14]. However, the role of PU.1 in regulating the transcription and expression of hDectin-1 gene during *A. fumigatus* stimulation in THP-1 cells remains unknown. In our study, the results of the luciferase reporter assay showed that PU.1 promoted hDectin-1 gene activity in a dose-dependent manner. Additionally, five potential TFBSs for PU.1 were predicted by using the TFBS databases. Moreover, the ChIP assay in our study indicated that PU.1 could bind to the hDectin-1 gene promoter at the –1053 to –1047, –719 to –713, and –527 to –521 regions. The synthetic polymer poly(dI-dC) is considered to be one of the most potent, and likely the most widely used, nonspecific competitor in EMSA. Its addition to the reaction mixture before addition of crude nuclear proteins has been proven to be an efficient way of reducing any nonspecific interactions by facilitating detection of the complexes of interest [39]. Therefore, we used poly(dI-dC) in the EMSA analysis in this study. Further EMSA results confirmed that PU.1 could bind to the abovementioned three regions of the hDectin-1 gene promoter. Based on the results of our study and those of previous reports, we suggest that PU.1 can bind to the hDectin-1 gene promoter.

To confirm that PU.1 regulates the expression of the hDectin-1 gene, we knocked down the expression of PU.1 in THP-1 cells using siRNA. Although Dectin-1 expression has been reported to be controlled by PU.1 [15] and leukotriene B4 via a GM-CSF/PU.1 axis [14] in mouse models, our results are the first to demonstrate that silencing the expression of PU.1 could significantly decrease the hDectin-1 gene mRNA and protein expression levels in THP-1 cells. Lack of PU.1 expression is known to contribute to decreased maturation, differentiation, and surfactant metabolism in AMs of patients with pulmonary alveolar proteinosis [40]. In addition, PU.1 knockdown in macrophages has shown to decrease phagocytic activity [15]. However, further studies are warranted to examine how knockdown or overexpression of PU.1 can affect the innate immune and inflammatory responses to *A. fumigatus* infection and the precise mechanisms underlying this process.

## Conclusions

As Dectin-1 is a major recognition receptor for numerous fungi, including species of *Candida, Aspergillus, Histoplasma, Cryptococcus,* and *Coccidioides* [41]*,* our data suggest a potentially broad role for PU.1 in antifungal defense. Meanwhile, Dectin-1 may participate in recognizing other microbes as well, and it may therefore be important that future studies investigate the potential roles of PU.1 in defense against infections by other pathogens. In conclusion, this study provides novel insights to modulate the immune response to *A. fumigatus*, and lays a foundation for the diagnosis and therapeutic strategy for *A. fumigatu*s aspergillosis.

## Abbreviations

BSA, Bovine serum albumin; ChIP, Chromatin immunoprecipitation; CLR, C-type lectin receptor; DAPI, 4′,6-Diamidino-2-phenylindole; DMEM, Dulbecco’s modified Eagle’s medium; EMSA, Electrophoretic mobility shift assays; ETS, E-twenty-six; FBS, Fetal bovine serum; HBE, Human bronchial epithelial; IA, Invasive aspergillosis; MOI, Multiplicity of infection; NSB, Nonspecific binding; PAGE, Polyacrylamide gel electrophoresis; PAMP, Pathogen-associated molecular pattern; PBS, Phosphate-buffered saline; PBST, Phosphate-buffered saline (PBS)-0.1 % (vol/vol) Tween 20; PCR, Polymerase chain reaction; PMA:Phorbol-12-myristate-13-acetate; PRR, Pattern recognition receptor; PVDF, Polyvinylidene difluoride; RT, Reverse transcription; SDS, Sodium dodecyl sulfate; TFBS, Transcription factor-binding site; UTR, Untranslated region
